# Supplementary material for: Language Disorder in Progressive Supranuclear Palsy and Corticobasal Syndrome: Neural Correlates and Detection by the MLSE Screening Tool
Source: Front Aging Neurosci. 2021 Jul 19;13:675739. doi: 10.3389/fnagi.2021.675739 (PMC8351757; doi:10.3389/fnagi.2021.675739)
Supplement: SUPPLEMENTARY TABLE 1 — Comparison between patients who were or were not included in the MRI analysis. [file Table_1.docx]

**Supplementary Table 1.** Comparison between patients who were or were not included in the MRI analysis

|  | **Included** | **Not included** | ***P*** |
| --- | --- | --- | --- |
| **N** | 38 | 13 | - |
| **Sex (M/F)** | 18/20 | 8/5 | .575 |
| **Age**  **Mean (SD)** | 69.84 (6.81) | 70.85 (7.43) | .705 |
| **Education (years)**  **Mean (SD)** | 12.11 (2.28) | 12.54 (2.54) | .592 |
| **Symptom duration (months)**  **Mean (SD)** | 38.50 (27.24) | 57.46 (52.50) | .391 |
| **ACE-III score**  **Mean (SD)** | 75.11 (16.13) | 50.31 (20.54) | **<.001** |
| **BDAE composite**  **Mean (SD)** | 47.17 (6.59) | 35.42 (12.25) | **<.001** |
| **MLSE total score**  **Mean (SD)** | 79.92 (16.95) | 63.15 (21.83) | **.021** |

Note: *P* values are the result of Chi squared, Welch’s ANOVA, or Kruskal-Wallis rank sum tests for each row

**Supplementary Table 2.** The Structure-LV loading values of each structure (from the Desikan-Killiany atlas) and subcortical volumes, arranged from highest to lowest

| **Structure Name** | **Structure-LV loading** |
| --- | --- |
| L Amygdala volume | 0.573 |
| L Superior Temporal thickness | 0.510 |
| L Caudal Middle Frontal thickness | 0.505 |
| L Putamen volume | 0.488 |
| R Entorhinal thickness | 0.466 |
| L Fusiform thickness | 0.455 |
| L Entorhinal thickness | 0.436 |
| L Hippocampus volume | 0.431 |
| L Precentral thickness | 0.429 |
| L Pars Opercularis thickness | 0.414 |
| L Middle Temporal thickness | 0.414 |
| R Insula thickness | 0.414 |
| R Precentral thickness | 0.412 |
| R Superior Temporal thickness | 0.406 |
| R Pars Opercularis thickness | 0.402 |
| L Insula thickness | 0.400 |
| R Lateral Orbitofrontal thickness | 0.397 |
| L Caudate volume | 0.396 |
| R Putamen volume | 0.392 |
| R Caudal Middle Frontal thickness | 0.392 |
| L Temporal Pole thickness | 0.390 |
| L Inferior Temporal thickness | 0.381 |
| R Hippocampus volume | 0.372 |
| L Pars Triangularis thickness | 0.363 |
| L Rostral Anterior Cingulate thickness | 0.360 |
| R Fusiform thickness | 0.359 |
| L Superior Frontal thickness | 0.358 |
| L Medial Orbitofrontal thickness | 0.333 |
| R Caudate volume | 0.329 |
| R Posterior Cingulate thickness | 0.323 |
| L Nucleus Accumbens volume | 0.314 |
| R Amygdala volume | 0.309 |
| R Temporal Pole thickness | 0.305 |
| L Posterior Cingulate thickness | 0.299 |
| R Superior Frontal thickness | 0.296 |
| R Nucleus Accumbens volume | 0.294 |
| L Transverse Temporal thickness | 0.294 |
| L Rostral Middle Frontal thickness | 0.291 |
| R Medial Orbitofrontal thickness | 0.283 |
| R Parahippocampal thickness | 0.282 |
| R Transverse Temporal thickness | 0.281 |
| R Thalamus volume | 0.278 |
| R Banks of Superior Temporal Sulcus thickness | 0.277 |
| L Lateral orbitofrontal thickness | 0.275 |
| L Supramarginal thickness | 0.270 |
| L Thalamus volume | 0.260 |
| R Precuneus thickness | 0.252 |
| L Paracentral thickness | 0.243 |
| R Middle Temporal thickness | 0.243 |
| R Rostral Middle Frontal thickness | 0.239 |
| R Pars Triangularis thickness | 0.221 |
| R Inferior Temporal thickness | 0.207 |
| L Pars Orbitalis thickness | 0.204 |
| R Frontal Pole thickness | 0.202 |
| R Supramarginal thickness | 0.198 |
| R Isthmus Cingulate thickness | 0.196 |
| L Parahippocampal thickness | 0.195 |
| L Precuneus thickness | 0.194 |
| R Paracentral thickness | 0.185 |
| R Pars Orbitalis thickness | 0.178 |
| R Inferior Parietal thickness | 0.177 |
| L Banks of Superior Temporal Sulcus thickness | 0.174 |
| L Pallidum volume | 0.148 |
| Brainstem | 0.144 |
| L Frontal Pole thickness | 0.134 |
| L Lingual thickness | 0.098 |
| L Superior Parietal thickness | 0.084 |
| L Isthmus Cingulate thickness | 0.079 |
| R Superior Parietal thickness | 0.076 |
| R Rostral Anterior Cingulate thickness | 0.071 |
| R Pallidum volume | 0.060 |
| R Lateral Occipital thickness | 0.036 |
| R Lingual thickness | 0.004 |
| L Postcentral thickness | 0.003 |
| L Caudal Anterior Cingulate thickness | -0.003 |
| R Postcentral thickness | -0.008 |
| L Inferior Parietal thickness | -0.009 |
| R Pericalcarine thickness | -0.016 |
| R Caudal Anterior Cingulate thickness | -0.016 |
| L Lateral Occipital thickness | -0.038 |
| L Pericalcarine thickness | -0.050 |
| R Cuneus thickness | -0.052 |
| L Cuneus thickness | -0.202 |
